# Supplementary material for: HIVprotI: an integrated web based platform for prediction and design of HIV proteins inhibitors
Source: J Cheminform. 2018 Mar 9;10:12. doi: 10.1186/s13321-018-0266-y (PMC5845081; doi:10.1186/s13321-018-0266-y)
Supplement: Supplementary file 2 — Additional file 2. Source code of HIVProtI web server. [file 13321_2018_266_MOESM2_ESM.zip › HIVprotI_Source-code/hivprotI/weka-3-6-6/doc/weka/gui/FileEditor.html]

FileEditor


---


|  |  |  |
| --- | --- | --- |
| **Overview** \| **Package** \| **Class** \| **Tree** \| **Deprecated** \| **Index** \| **Help** \| **Weka's home** | |  |
| **PREV CLASS**   **NEXT CLASS** | **FRAMES**    **NO FRAMES**     **All Classes** |  |
| SUMMARY: NESTED \| FIELD \| CONSTR \| METHOD | DETAIL: FIELD \| CONSTR \| METHOD |  |


---


## weka.gui Class FileEditor

```
java.lang.Object
  java.beans.PropertyEditorSupport
      weka.gui.FileEditor
```

**All Implemented Interfaces:**: java.beans.PropertyEditor

---

``` public class FileEditor extends java.beans.PropertyEditorSupport ```

A PropertyEditor for File objects that lets the user select a file.

**Version:**
:   $Revision: 7059 $

**Author:**
:   Len Trigg (trigg@cs.waikato.ac.nz)

---

**Constructor Summary**

`FileEditor()`


| **Method Summary** | |
| --- | --- |
| `java.awt.Component` | `getCustomEditor()`             Gets the custom editor component. |
| `java.lang.String` | `getJavaInitializationString()`             Returns a representation of the current property value as java source. |
| `boolean` | `isPaintable()`             Returns true since this editor is paintable. |
| `void` | `paintValue(java.awt.Graphics gfx, java.awt.Rectangle box)`             Paints a representation of the current Object. |
| `boolean` | `supportsCustomEditor()`             Returns true because we do support a custom editor. |

**Methods inherited from class java.beans.PropertyEditorSupport**

`addPropertyChangeListener, firePropertyChange, getAsText, getSource, getTags, getValue, removePropertyChangeListener, setAsText, setSource, setValue`

**Methods inherited from class java.lang.Object**

`equals, getClass, hashCode, notify, notifyAll, toString, wait, wait, wait`

**Constructor Detail**

### FileEditor

```
public FileEditor()
```


**Method Detail**

### getJavaInitializationString

```
public java.lang.String getJavaInitializationString()
```

:   Returns a representation of the current property value as java source.

    :   **Specified by:**: `getJavaInitializationString` in interface `java.beans.PropertyEditor` **Overrides:**: `getJavaInitializationString` in class `java.beans.PropertyEditorSupport`
    :   **Returns:**: a value of type 'String'

---


### supportsCustomEditor

```
public boolean supportsCustomEditor()
```

:   Returns true because we do support a custom editor.

    :   **Specified by:**: `supportsCustomEditor` in interface `java.beans.PropertyEditor` **Overrides:**: `supportsCustomEditor` in class `java.beans.PropertyEditorSupport`
    :   **Returns:**: true

---


### getCustomEditor

```
public java.awt.Component getCustomEditor()
```

:   Gets the custom editor component.

    :   **Specified by:**: `getCustomEditor` in interface `java.beans.PropertyEditor` **Overrides:**: `getCustomEditor` in class `java.beans.PropertyEditorSupport`
    :   **Returns:**: a value of type 'java.awt.Component'

---


### isPaintable

```
public boolean isPaintable()
```

:   Returns true since this editor is paintable.

    :   **Specified by:**: `isPaintable` in interface `java.beans.PropertyEditor` **Overrides:**: `isPaintable` in class `java.beans.PropertyEditorSupport`
    :   **Returns:**: true.

---


### paintValue

```
public void paintValue(java.awt.Graphics gfx,
                       java.awt.Rectangle box)
```

:   Paints a representation of the current Object.

    :   **Specified by:**: `paintValue` in interface `java.beans.PropertyEditor` **Overrides:**: `paintValue` in class `java.beans.PropertyEditorSupport`
    :   **Parameters:**: `gfx` - the graphics context to use: `box` - the area we are allowed to paint into


---


|  |  |  |
| --- | --- | --- |
| **Overview** \| **Package** \| **Class** \| **Tree** \| **Deprecated** \| **Index** \| **Help** \| **Weka's home** | |  |
| **PREV CLASS**   **NEXT CLASS** | **FRAMES**    **NO FRAMES**     **All Classes** |  |
| SUMMARY: NESTED \| FIELD \| CONSTR \| METHOD | DETAIL: FIELD \| CONSTR \| METHOD |  |


---
